# Prevalence and Antibiotic Resistance of *Salmonella* and *Campylobacter* Isolates from Raw Chicken Breasts in Retail Markets in the United States and Comparison to Data from the Plant Level

**DOI:** 10.3390/life13030642

**Published:** 2023-02-25

**Authors:** Sana Mujahid, Michael Hansen, Robyn Miranda, Keith Newsom-Stewart, James E. Rogers

**Affiliations:** Consumer Reports, 101 Truman Avenue, Yonkers, NY 10703, USA

**Keywords:** *Salmonella*, *Campylobacter*, antibiotic resistance, retail chicken

## Abstract

Chicken is the most popular meat in the United States, and consumers may be exposed to multidrug resistant *Salmonella* and *Campylobacter* through consumption of retail chicken breasts. This study aimed to (i) determine the percentage of raw, packaged, retail chicken breasts from 27 metro areas that tested positive for *Salmonella* and *Campylobacter*; (ii) investigate the antibiotic susceptibility profiles of a subset of the isolates; and (iii) compare the *Salmonella* prevalence data to establishment level *Salmonella* categorization data published by the U.S. Department of Agriculture (USDA). USDA Food Safety and Inspection Service (FSIS) Microbiology Laboratory Guidebook (MLG) methodology was used to isolate and identify *Salmonella* (*n* = 672), *Campylobacter* (*n* = 499) from 400 g samples. National Antimicrobial Resistance Monitoring System (NARMS) methodology was followed for antimicrobial susceptibility testing of *Salmonella* (*n* = 52) and *Campylobacter* (*n* = 16) isolates. *Salmonella* was found in 8.6% of samples and *Campylobacter* in 4.2%. Having a 3 rating in USDA’s *Salmonella* Categorization of Individual Establishments for chicken parts was predictive of having a higher *Salmonella* percent positive in our data set (*p* ≤ 0.05). A total of 73.1% of *Salmonella* isolates, and 62.5% of *Campylobacter* isolates were resistant to ≥one class of antibiotics, with 48.1% of *Salmonella* isolates resistant to ≥three classes. Current results support interventions that take a ‘farm-to-fork’ approach with distinction by poultry types and parts as well as serovars, to lower antibiotic resistant *Salmonella* infections in humans due to poultry. **Highlights:** *Salmonella* was found in 8.6% and *Campylobacter* in 4.2% of chicken breasts tested; A 3 rating by USDA was predictive of a higher *Salmonella* percent positive; 48.1% of *Salmonella* isolates were resistant to 3 or more classes of antibiotics.

## 1. Introduction

Raw poultry products frequently contain pathogenic organisms such as *Salmonella* and *Campylobacter*, both of which are among the top five pathogens contributing to domestically acquired foodborne illnesses, hospitalizations, and deaths [1]. The severity of infections and the public health burden from foodborne bacteria, such as *Salmonella* and *Campylobacter*, can be much greater when these bacteria are antibiotic resistant [2]. 

During 2021, most infections reported to FoodNet were caused by *Campylobacter* or *Salmonella*, with the five most common *Salmonella* serotypes for more than 10 years remaining predominant [3]. Contamination with these pathogens can occur at multiple steps along the food chain, including on-farm production, processing, distribution, retail marketing, and during handling or preparation [4,5]. Food poisoning symptoms most often caused by these foodborne pathogens include stomach cramps, fever, vomiting and diarrhea; however, symptoms that are more serious may include bloody diarrhea and temporary paralysis [6,7]. Many animal species serve as reservoirs for these pathogens, especially chickens. According to CDC outbreak data from 2009–2015, chicken was responsible for the most illnesses, sickening 3114 people, or about 12% of the total illnesses, in the outbreaks for which a specific food was determined to be the cause [8]. Foodborne illness source attribution estimates for 2019 indicate that 64.7% of non-dairy *Campylobacter* illnesses were attributed to chicken [9]. Microbiological testing of raw meat purchased at the retail level provides information on possible consumer exposure to pathogens on raw meat prior to the food handling and preparation stages, as well as potential cross-contamination risk in commercial facilities and domestic settings.

Many of the isolated *Salmonella* and *Campylobacter* that can cause disease have displayed antimicrobial resistance phenotypes. Antimicrobial agents such as fluoroquinolones and the cephalosporin class of antibiotics are the drugs of first choice for the treatment of adult salmonellosis; however, in recent years an increased number of antibiotic resistant *Salmonella* strains have been isolated from human cases [10,11,12]. In a 2021 report, Medalla et al. estimated a 40% increase in the annual incidence of non-typhoidal *Salmonella* (NTS) infections with clinically important resistance in the United States, with approximately 222,000 infections in 2015–2016 compared with around 159,000 in 2004–2008. Mukherjee et al., 2019 found an increase in the frequency of tetracycline resistant and multidrug resistant (MDR) NTS infections from 2011 to 2014 in the state of Michigan. In 2020, 17 multistate outbreaks of *Salmonella* illnesses were linked to contact with poultry in backyard flocks. There were 1722 cases, 333 hospitalizations and 1 death. Whole genome sequencing of *Salmonella* isolates from 1641 ill people and 2 environmental samples was performed and indicated antibiotic resistance among 848 isolates, including some resistant to more than one antibiotic [13]. The National Antimicrobial Resistance Monitoring System for Enteric Bacteria (NARMS), a collaborative effort of the CDC, FDA, USDA, and state/local public health departments, found approximately 17% of *Salmonella* isolates from retail chicken to be multidrug resistant in 2017 [14].

Antibiotics are used in human and veterinary medicine to treat and prevent disease, and for other purposes including growth promotion of food animals. Use of medically important antimicrobials (MIAs) is not permitted for growth promotion use, while antibiotics not considered medically important, such as bacitracin, can still be used for growth promotion [15,16,17]. Research shows that use of bacitracin in food animals may also select for resistance to colistin, a ‘last resort’ antibiotic for treatment of MDR-resistant infections in humans [18]. Although the World Health Organization (WHO) designates bacitracin as an MIA, the FDA does not consider it one [19,20]. Widespread use of antibiotics in animals raised for food, and the possibility of subsequent contamination of food with MDR resistant bacteria poses a threat to public health, as this bacteria of animal origin can be transmitted to humans through several pathways, including inadequate cooking and mishandling of food [21]. The recall of approximately 40,000 pounds of rotisserie chicken products in 2013 in the United States due to MDR *Salmonella* Heidelberg underscores the gravity of the public health risk and the economic damage posed by MDR *Salmonella* in food products of animal origin. In total, 634 cases were reported from 29 different states and included infection with 7 different strains of *Salmonella* Heidelberg [22,23]. 

Chicken is the most popular meat in the U.S., with higher consumption per capita than beef, pork, or turkey [24]. This study focused on products derived from chicken breasts as they represent the highest percentage of sales among chicken parts in the U.S. [25]. The purpose of the first phase of this study was to determine the percentage of raw, packaged, retail chicken breasts that test positive for *Salmonella* and *Campylobacter* and compare the results to regulatory data at the plant level. In the second phase of this study, we investigated the antibiotic susceptibility profiles of a subset of the *Salmonella* and *Campylobacter* isolates. 

## 2. Materials and Methods

### 2.1. Samples

Products tested in this study were pre-packaged, fresh (not frozen), raw, chicken breast derived products sold at retail markets. The following chicken breast types were included: (1) Boneless Tenders/Skinless, (2) Boneless Regular/Skinless, (3) Boneless Thin Sliced/Skinless, (4) Bone-in Split/Skin-on, and (5) Bone-in Split/Skinless. Samples of the 5 major chicken brands in the United States (as determined by the share of chicken breast sales) were purchased, as well as store brands, private labels, clear film, including organic and ‘no antibiotics’ samples. The following were not included in the study: fresh ground chicken or other meat; pre-seasoned (i.e., marinated) chicken products; chicken sold in butcher cases (all chicken was pre-packaged); pre-cooked or ready-to-eat chicken.

A pre-retrieval survey was conducted by a contract retrieval firm. A total of twenty-seven metro areas, approximately 3 from each of 9 census sub-regions/divisions, were included in the survey. A shopper visited approximately 7 different stores—~2 in the center city, ~3 in the suburbs, and ~2 in outer suburbs—to determine the availability of pre-packaged brands of fresh, raw chicken breasts. Of the approximately 7 stores, ~4 were different supermarket chains, and ~1 was a gourmet or natural/health food-type store. The remaining ~2 stores were either a club/big box store, or if none of those were available, 2 other supermarket chain stores. Based on the data from the pre-retrieval survey, a sample retrieval design was developed to purchase products in approximately the same proportions found in the pre-retrieval survey from the 27 metro areas; except that the five major brands were oversampled to reflect their higher market share. Samples were further distributed among the five major brands—conventional, non-major brands—conventional, 5 major brands—no antibiotics claim, and non-major brands—no antibiotics claim. 

Samples with the ‘No Antibiotics’ claim were divided into ‘verified’ and ‘unverified’ categories. A ‘no antibiotics’ claim was considered verified if one or more of the following seals also appeared on the label- USDA Organic, Organic, USDA Process Verified; or if the brand name was ‘Whole Foods’ or ‘365 Everyday Value’. The USDA requires that the “raised without antibiotics” or similar claims mean no antibiotics at any stage of life, including in ovo administration (this standard applies to both verified and unverified “no antibiotics” claims); however, organic production allows chickens to have had antibiotics (usually gentamicin) injected into the eggs before they hatch and on the first day of the life of the chick. If the “raised without antibiotics” claim appears on chicken labeled “organic,” the USDA standard for “raised without antibiotics” or similar claims overrides the organic exemption. 

Packaged chicken breast samples were obtained by the retrieval firm in January and February 2018. Upon being purchased, all samples were blind coded, placed into insulated shipping containers, and packed with cold packs and a one-time use temperature monitoring device (Sensitech, Inc., Beverly, MA, USA). The temperature logger was started immediately before sealing the box and was set to continuously record the temperature during the shipment process. 

The samples were shipped overnight to a contract laboratory with the intention of having all samples arrive on the day after sample purchase to preserve sample temperature. However, provisions were made to accept samples that arrived two days after the sample purchase date as long as the sample temperature remained ≤10 °C.

### 2.2. Sample Processing

All samples analyzed by the laboratory met temperature, package integrity, and labeling criteria. Samples were discarded if: (1) the sample was outside the acceptable temperature range (>10 °C upon receipt or spike during shipment); (2) sample integrity was compromised (e.g., package ruptured, leaking, spoiled); (3) the sample seal was not intact; (4) the sample collection date on the shipping form was more than two days prior to receipt; or (5) the samples were unlabeled or mislabeled. 

Samples that passed the above acceptance criteria underwent further processing. Each package was weighed to determine its bag weight, and the total number of pieces in each package were counted. Each package was then aseptically opened and approximately 400 g of the chicken parts were sub-sampled from the package. This 400 g sub-sample was then placed into a sterile Whirl-Pak bag for further processing. All remaining pieces in a package were kept in storage at 4 °C. 

To prepare the chicken parts for analysis, 400 mL of Buffered Peptone Water (BPW; Becton, Dickinson and Company, Sparks, MD, USA) was added to each ~400 g sub-sample. Each sub-sample was set as close to 400 g as possible without breaking apart the various pieces of chicken parts. Each sample was then rinsed with a rocking motion for one minute (ca. 35 RPM). This was done by grasping the parts in the bag with one hand and the closed top of the bag with the other. The samples were then rocked with a reciprocal motion in about an 18–24 inch arc, assuring that all surfaces were rinsed. This rinsate was then used for all further testing. The Whirl-Pak bag with the chicken parts and rinsate was then retained at 4 °C until all testing was complete. USDA/FSIS MLG (Microbiology Laboratory Guidebook) [26] methodology was used to isolate and identify *Salmonella* and *Campylobacter* from the ~400 g chicken samples. 

### 2.3. Campylobacter spp. Analysis

For *Campylobacter* spp. analysis (*n* = 499), each BPW poultry parts rinsate was set at a 1:1 dilution (30 mL of rinsate with 30 mL of Double-Strength Bolton Broth (Remel, Inc.; Lenexa, KS, USA) with Supplement (Remel, Inc.)) and was incubated under microaerophilic conditions in a vented culture flask at 42 ± 1 °C for 24–48 h. Following incubation, samples were run on the BAX^®^ Real-Time *Campylobacter* assay (Hygiena, LLC; Camarillo, CA, USA) and the enrichments in vented culture flasks were retained at 4 °C after testing. For BAX^®^ analysis, the lysis buffer was prepared following the manufacturer’s instructions. From the enriched sample bag, 5 μL of each sample was transferred into the prepared lysis tubes. The tubes were heated on a 37 °C heat block for 20 min and then on a 95 °C heating block for 10 min. The lysis tubes were then placed on a cooling block (2–8 °C) for 5 min. After the cooling period, 30 μL of the lysate was transferred to amp tubes. The Real-Time *Campylobacter* assay was chosen on the BAX^®^ software (Version 3.6 b6005, Hygiena, LLC; Camarillo, CA, USA), and amp tubes were loaded into the BAX^®^ Q7 instrument for PCR. Enrichments from samples that screened positive on the BAX^®^ were streak plated onto Campy-Cefex Agar (Neogen; Lansing, MI, USA) for isolation and were incubated under microaerophilic conditions at 42 ± 1 °C for 48 ± 2 h. Colonies exhibiting typical morphology for *Campylobacter* spp. on Campy-Cefex 

Agar (translucent or mucoid and glistening, flat or slightly raised, and pink or yellow-gray in color) were considered presumptive positive. Typical colonies were subjected to the Latex Agglutination Immunoassay (SCIMEDX Corporation; Denville, NJ, USA) for confirmation. Samples that had typical morphology on Campy-Cefex Agar and had a positive agglutination result with the Latex Agglutination Immunoassay were considered positive for *Campylobacter* spp. 

In addition to confirmation by latex agglutination, all *Campylobacter* spp. isolates were also subjected to biochemical confirmation with the VITEK^®^ 2 Compact NH ID Card (bioMérieux) to determine the species of the isolate. 

### 2.4. Salmonella spp. Analysis

For *Salmonella* spp. analysis (*n* = 672), each rinsate was set at a 1:1 dilution (30 mL of rinsate with 30 mL of BPW) and was incubated aerobically at 35 ± 1 °C for 20–24 h. Following incubation, the samples were analyzed using the BAX^®^ *Salmonella* 2 assay (Hygiena, LLC), and the enrichment bags were retained at 4 °C after testing. For BAX^®^ analysis, the lysis buffer was prepared following the manufacturer’s instructions. From the enriched sample bag, 5 μL of each sample was transferred into the prepared lysis tubes. The tubes were heated on a 37 °C heat block for 20 min and then on a 95 °C heating block for 10 min. The lysis tubes were then placed on a cooling block (2–8 °C) for 5 min. After the cooling period, 50 μL of the lysate was transferred to amp tubes. The *Salmonella* 2 assay was chosen on the BAX^®^ software, and amp tubes were loaded into the BAX^®^ Q7 instrument for PCR. Samples that screened positive on the BAX^®^ were confirmed following the USDA-MLG protocol. Briefly, 500 μL of enrichment from each positive-screened sample was added to 10 mL of Tetrathionate Hajna Broth (TT Hajna; Becton, Dickinson and Company) with 400 μL of TT Hajna Iodine Solution (Becton, Dickinson and Company). Likewise, 100 μL of sample was transferred into Modified Rappaport–Vassiliadis Broth (mRV, Becton, Dickinson and Company). The mRV and TT Hajna tubes were incubated at 42 ± 0.5 °C for 22–24 h in a dry incubator or at 42 ± 0.5 °C for 18–24 h in a water bath. After incubation, all mRV and TT Hajna tubes were streak plated to Brilliant Green Sulfa Agar (BGS, Becton, Dickinson and Company) and Xylose–Lysine–Tergitol 4 Agar (XLT4; Becton, Dickinson and Company) and incubated at 35 ± 2 °C for 18–24 h. Colonies exhibiting typical morphology on BGS (pink and opaque colonies) and XLT4 (black and red colonies with or without black centers) were considered presumptive positive. Typical colonies were struck to Triple Sugar Iron (TSI; Becton, Dickinson and Company) and Lysine Iron Agar (LIA; Becton, Dickinson and Company) slants, which were then incubated at 35 ± 2 °C for 24 ± 2 h. At least three total colonies were tested before calling a sample negative for *Salmonella* spp. Samples that gave typical reactions for *Salmonella* were confirmed using serological and biochemical testing. Serological testing was performed using Polyvalent A-I and Vi *Salmonella* O Antiserum (Becton, Dickinson and Company) for O serology testing and the *Salmonella* Latex Test (Oxoid, Ltd., Basingstoke, UK) for H serology testing. Agglutination of the sample was a positive result. Samples that displayed agglutination or auto-agglutination were tested with biochemical confirmation. Slants that displayed positive results with serological testing were streak plated to Tryptic Soy Agar (TSA; Becton, Dickinson and Company, Sparks, MD, USA) and incubated at 35 ± 2 °C for 18–24 h. The TSA plates were used for biochemical confirmation on the VITEK^®^ 2 Compact System with GN ID Cards (bioMérieux; Marcy-l’Étoile, France). The manufacturer’s instructions were followed for sample preparation and loading into the VITEK. Samples that were identified as *Salmonella* spp. on the VITEK^®^ 2 Compact GN ID Cards were considered positive. All biochemically confirmed *Salmonella* spp. isolates were further characterized via traditional Kaufman–White serotyping. 

### 2.5. Positive Controls and Duplicate Samples

Positive controls and duplicate samples were incorporated into the daily testing scheme to provide additional quality control. Approximately 5% of all samples that passed the receiving acceptance criteria provided were tested as duplicate samples. This equated to roughly every 20th sample being tested as a duplicate sample. In order to create a duplicate sample, two ~400 g sub-samples were obtained from the same package. The duplicate samples were processed alongside all other samples. 

### 2.6. Retain Procedures for Positive Isolates

All positive isolates that were obtained were immediately prepared for long term retain storage at −80 °C. For *Campylobacter* spp., positive isolates from Campy–Cefex Agar were grown in Double-Strength Bolton Broth with supplement. For *Salmonella* spp., positive isolates from TSA plates were grown in Tryptic Soy Broth (TSB; Becton, Dickinson and Company). In all cases, an 850 μL aliquot of the broth culture of each organism was then added to a cryogenic storage vial that was pre-filled with 150 μL of sterilized glycerol; the concentration of glycerol in the final solution was 15%. In certain instances, positive isolates had to be resuscitated from materials that were kept in refrigerated retain. In some cases, resuscitation procedures were unsuccessful in providing a final isolate to perform additional testing. 

### 2.7. Whole Genome Sequencing

Whole-genome sequencing (WGS) was performed for 19 *Salmonella* Infantis isolates. Sequencing was performed on the entire gDNA extracted from each sample. The genome library was prepared using the Nextera XT DNA sample prep kit (Illumina, CA, USA), and genome sequencing was performed using the Illumina MiSeq desktop sequencer (Illumina) loaded with a paired-end 2 × 250 cycle MiSeq reagent kit version 3. The raw sequencing reads were quality filtered using the default settings of “fastp” software [27]. The filtered reads were then assembled using the SPAdes assembler https://cab.spbu.ru/software/spades/ (SPAdes 3.13.1). The resulting contigs were then used for preliminary identification using an in-house mash database [28]. From the whole-genome assembly, presence or absence of various virulence, typing and antibiotic resistance genes was determined using SRST2 software (https://genomemedicine.biomedcentral.com/articles/10.1186/s13073-014-0090-6) (SRST2 v0.2.0). SISTR (https://github.com/phac-nml/sistr_cmd) (sistr_cmd v1.0.2) was used for serotype determination.

### 2.8. Antimicrobial Susceptibility Testing

A total of 52 *Salmonella* isolates and 16 *Campylobacter* isolates were analyzed for resistance to 14 antibiotics for *Salmonella,* and 8 antibiotics for *Campylobacter* (Tables S1 and S2). *Salmonella* isolates on semisolid media were subcultured twice onto trypticase soy agar/5% sheep (TSAB) blood to assure purity prior to antimicrobial susceptibility testing (AST) procedures. *Campylobacter* isolates were subcultured onto TSAB, incubated at 42 °C overnight in microaerophilic conditions, and subsequently banked into 10% sterile glycerol tubes and stored at −80 °C until testing could be performed. *Campylobacter* isolates from frozen stocks were passed twice on TSAB under microaerophilic conditions prior to AST.

Susceptibility testing was performed using methods prescribed by the latest edition (2016) of the National Antimicrobial Resistance Monitoring System (NARMS) Manual of Laboratory Methods [29] using the following consumables: Sensititre CMV3AGNF plate (*Salmonella*); Sensititre Cation-adjusted Mueller–Hinton Broth (*Salmonella*); Sensititre CAMPY2 plate (*Campylobacter*); Sensititre Mueller–Hinton Broth supplemented with lysed horse blood (*Campylobacter*); and Sensititre sterile water tubes. Interpretative breakpoints for susceptible (S), intermediate (I), and resistant (R) were applied based on current standards. “NI” was used to communicate “No interpretation” when none was available [30]. The 0.5 McFarland suspensions of isolates were generated using a Trek nephelometer with a Trek turbidity standard, and plates were inoculated using a Trek AIM autoinoculator. CMV3AGNF plates were incubated for 18 h at 35 °C in ambient air, and CAMPY plates were incubated for 24 h at 42 °C in microaerophilic conditions. All plates were manually read on a BIOMIC instrument using software version 7.9.1.2019 V3c Matrix. Not all the *Salmonella* and *Campylobacter* isolates obtained from the chicken breast samples could undergo AST as explained above. 

### 2.9. Statistical Analyses

Data were analyzed using GLIMMIX, LOGISTIC, and Base SAS from the SAS statistical software package (version 9.4). A logistic mixed effects model used the design factors listed in Section 2.1 to estimate prevalence for the individual organism *Salmonella* and the combination *Salmonella* and/or *Campylobacter*. Prevalence by brand, organic category, product type, plant, parent company, and USDA regulatory category was calculated. *Campylobacter* prevalence was not estimated by mixed models due to the very small number of positives; only the raw proportion of positive samples was calculated. USDA *Salmonella* categorization of plants was used to predict prevalence at those plants using logistic regression and chi-square analysis. The proportion of antibiotic resistant samples were obtained from raw counts or percentages. Adjusted values are model-based estimates, rather than raw proportions. Significance was set at *p*-value < 0.05 for all hypotheses tests.

## 3. Results and Discussion

**Figure 1:**
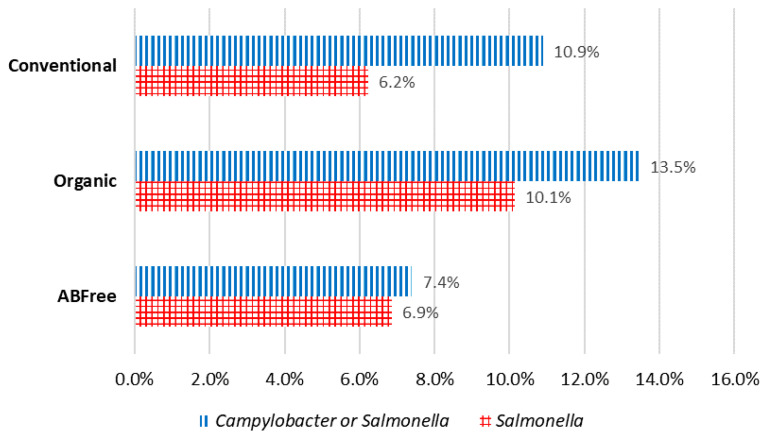
Levels of contamination *. * All values in Figure 1 are adjusted.

Overall Prevalence and Levels of Contamination by Brand or Category: *Campylobacter* was found in 4.2% of the chicken breasts and *Salmonella* was found in 8.6% of the chicken breasts. Of the major brands (Brand 1–Brand 5), Brand 1 had a significantly higher percent positive for *Salmonella* than all the other major brands. 0.6% of chicken breasts contained both *Salmonella* and *Campylobacter* of those tested for both, while 89.18% of chicken breasts were free of both *Salmonella* and *Campylobacter*. A total of 10.82% of chicken breasts contained one or both *Salmonella* and *Campylobacter* (*Salmonella* AND/OR *Campylobacter*), of those tested for both. No significant differences were found for the pathogens when the following categories were compared: (1) Non-organic unverified Antibiotic (AB)-free, Non-organic verified AB-free, Organic verified AB-free, and Conventional; (2) AB-free, Organic, and Conventional; (3) No AB-free claim and AB-free claim. Of the major brands, Brand 1 also had a significantly higher percent positive for *Salmonella* and/or *Campylobacter* than all the other major brands (Figure 1). NARMS retail chicken meat testing in 2015 found 6.1% of samples positive for *Salmonella* and 24% positive for *Campylobacter*, both showing a declining trend when compared to previous years [31]. The USDA’s baseline study on raw chicken parts obtained from processing plants in 2012 found positive rates of 26.3% for *Salmonella* and 21.4% for *Campylobacter* [32]. However, these studies had differing sample designs, collection points/methodologies, collection seasons, product types, and/or testing methodologies; and the results are not directly comparable. In addition, USDA FSIS published a study showing possible antimicrobial carryover in the rinsate from samples collected at the processing plant, causing false negative results [33]. 

Levels of Contamination by Parent Company and Plant: Through analysis of plant numbers found on sample packages, we found that at least 70% of samples, including store brands, private labels, and clear film samples, were sourced from five parent companies at the time of analysis. Among parent companies, Brand 1 had a significantly higher percent positive of *Salmonella* than other companies did. Brand 1 and Brand 2 had a significantly higher percent positive of *Campylobacter* and/or *Salmonella* than Brand 3 and Brand 4. Five of the six plants with the highest percent positive of *Salmonella* were Brand 1 plants, by ranking. 

**Figure 2:**
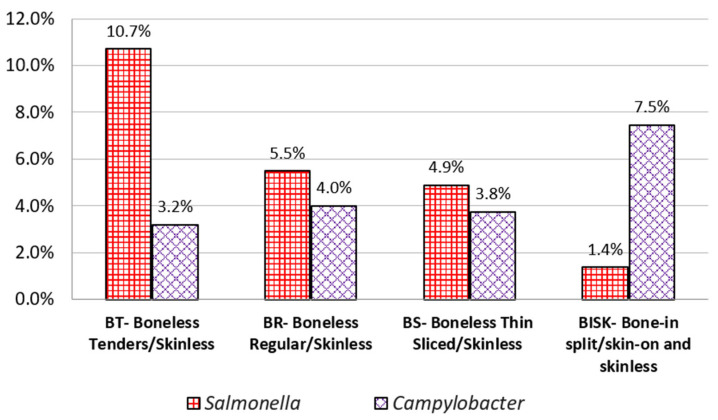
Levels of contamination by product type.

Levels of Contamination by Product Type: The data set only included two BIS (Bone-in-split/skinless) samples so they were merged with BISK (Bone-in-split/skin-on) samples, for this analysis. Boneless tenders/skinless (BT) had a significantly higher percent positive rate of *Salmonella* than BISK. Bone-in-Split/skin-on and skinless (BISK) samples had a significantly lower percent positive for *Salmonella* than tenders (BT) and Boneless regular/skinless (BR). Boneless thin sliced/skinless (BS) had a *Salmonella* percent positive closer to BR, but not statistically significantly different from BISK (Figure 2). Location and urbanicity were not found to be statistically significant in our modeling, so no further analyses (e.g., prevalence) were done for those factors.

Regulatory Results and Study Results Agree: USDA FSIS has established pathogen reduction performance standards, which it uses to evaluate an establishment’s food safety performance. As part of these standards, USDA FSIS conducts *Salmonella* categorization of individual establishments (plants) for poultry products, including ‘Chicken Parts’, and posts the results online. The establishments are placed into three categories, which are defined as follows [34]: Category 1: Establishments that have achieved 50 percent or less of the maximum allowable percent positive during the most recent completed 52-week moving window (meets the performance standard); Category 2: Establishments that meet the maximum allowable percent positive but have results greater than 50 percent of the maximum allowable percent positive during the most recent completed 52-week moving window (meets the performance standard); Category 3: Establishments that have exceeded the maximum allowable percent positive during the most recent completed 52-week moving window (does not meet the performance standard and must take corrective action); NA: FSIS did not collect or analyze the minimum number of samples to categorize the establishment and the establishment has not exceeded the maximum number of positives allowed under the standard [35]. The public disclosure of USDA *Salmonella* establishment categorization was found to correlate with a reduction in *Salmonella* levels, when data over a 4 year period (2006–2010) was analyzed, with establishments having poor or mediocre performance in one year improving their performance the following year [36].

In both comparisons to USDA data sets [(1) 26 November 2017–24 November 2018 and (2) 1 July 2018–29 June 2019], we found that having a category 3 rating, which is given to establishments that have exceeded the maximum allowable *Salmonella* percent positive, is predictive of having a high percent positive of samples for *Salmonella* in our data set; and that is not just due to outlier plants (Table 1). Our finding of a high percent of positive samples for *Salmonella* in Brand 1 products is supported by the USDA’s *Salmonella* rating for the Brand 1 plants that were in our data set.

Serotypes and Species: Of the *Salmonella* isolates serotyped, 19 were *S. Infantis*, 17 were *S. Typhimurium*, 10 were *S. Enteritidis*, and 6 were *S. Kentucky*. Of the *Campylobacter* isolates identified by speciation, eight were *Campylobacter coli* and eight were *Campylobacter jejuni*. Approximately 78% of the *S. Infantis* isolates identified were from Brand 1 plants. 

Sequences resulting from WGS of the 19 *Salmonella* Infantis isolates identified in this study were uploaded to the NCBI (National Center for Biotechnology Information) public database. 

Antibiotic Susceptibility Testing: The antibiotics used for susceptibility testing of the *Salmonella* and *Campylobacter* isolates and their CLSI classes are listed in Supplemental Tables S1 and S2. The CDC defines multidrug-resistant organisms (MDROs) as microorganisms that are resistant to one or more classes of antimicrobial agents [37]. NARMS defines multidrug resistance (MDR) as resistance to three or more antimicrobial classes [38]. In this study, we use the NARMS definition and consider an organism multidrug resistant when it is resistant to three or more classes of antibiotics. A total of five out of 52 *Salmonella* isolates tested were resistant to only one antibiotic; ten isolates were found to be resistant to two antibiotics; and 25 were resistant to three or more antibiotics. There were 12 *Salmonella* isolates that were not resistant to any antibiotics [Table 2]. Of the 52 *Salmonella* isolates tested, 76.92% were resistant to at least one class of antibiotics, and 48.08% were multidrug resistant. A total of five out of 16 *Campylobacter* isolates were resistant to only one antibiotic; three isolates were found to be resistant to two antibiotics; and two were resistant to three or more antibiotics. There were six *Campylobacter* isolates that were not resistant to any antibiotics. Of the 16 Campylobacter isolates tested, 50% were resistant to one class of antibiotics, 12.5% were resistant to two classes, and none were multidrug resistant [Table 3]. 

Antibiotics recommended by the CDC to treat *Salmonella* infections in humans include fluoroquinolones (for example, ciprofloxacin) for adults, azithromycin for children, and third generation cephalosporins such as ceftriaxone, which is recommended as an alternative first-line treatment agent [10,39]. Other antibiotics used to treat *Salmonella* infections include trimethoprim-sulfamethoxazole (TMP-SMZ) and ampicillin; however, resistance to these antibiotics is common [40], and our study found that 30.76% of the isolates were resistant to ceftriaxone and 32.69% were resistant to ampicillin. Our study found six *Salmonella* isolates that were resistant to three of the five recommended antibiotics for treating infections in humans, and ten isolates that were resistant to two of the five recommended antibiotics. There would thus only be a limited number of treatment options for infections caused by these isolates (Supplemental Tables S3 and S4).

Antibiotics such as azithromycin, (a macrolide) and ciprofloxacin (a fluoroquinolone) are used to treat *Campylobacter* infections. However, resistance to fluoroquinolones is common, initially being linked to the FDA’s approval of fluoroquinolones for use in poultry; the approvals have now been withdrawn by the FDA. Macrolides are thus the antibiotics of choice [41,42]. Our study found that none of the *Campylobacter* isolates were resistant to azithromycin, and 31.25% were resistant to ciprofloxacin (Supplemental Table S4). 

The AST data obtained from our 52 *Salmonella* isolates from 2018 were compared to the 2016–2017 NARMS data, which are the most recent NARMS data available [38]. The 2016–2017 NARMS Integrated Summary provides consolidated data from human clinical isolates, food-producing animal isolates from cecal (intestinal) samples at slaughter, samples collected at slaughter as part of Pathogen Reduction/Hazard Analysis Critical Control Point (PR/HACCP) testing, and raw retail meats (chicken, ground turkey, ground beef, and pork chops) collected at retail outlets in 18 states. The 2016–2017 NARMS chicken data is from retail chickens, PR/HACCP, and cecal samples [38]. 

In our data, 76.92% (40 of 52) of the *Salmonella* isolates were resistant to one or more classes of antibiotics, while 48.08% (25 of 52) were resistant to three or more classes of antibiotics (MDR). According to the 2016–2017 NARMS data, MDR levels of *Salmonella* in routinely sampled chickens went from 9.5% in 2015 to 18% in 2017, while MDR levels of *Salmonella* from chicken cecal samples went from 15% to 25%, making our numbers notably higher. For 2015, 2016 and 2017 the NARMS MDR levels in *Salmonella* isolates from retail chicken were 15.2%, 16.6% and 16.7%, respectively [14], as compared to 48.08% of our *Salmonella* isolates in 2018. 

NARMS data on ceftriaxone, which is recommended by the CDC as an alternative first-line treatment for *Salmonella* infections, show that resistance in *Salmonella* isolates from chicken samples collected routinely as part of PR/HACCP testing increased from 6.5% to 9.3%, for 2015 and 2017, respectively. In 2017, 11.1% of *Salmonella* isolates from retail chickens were resistant to ceftriaxone. Our data found ceftriaxone resistance in 30.8% (16 of 52) of the *Salmonella* isolates. Again, our results are notably worse.

Our data and NARMS found no resistance to ciprofloxacin in *Salmonella* isolates from retail chicken. However, NARMS also took data on DSC (decreased susceptibility), defined as isolates with a Minimum Inhibitory Concentration (MIC) *≥* 0.12 μg/mL. According to NARMS, DSC in *Salmonella* increased from <1% to 9% in retail chicken samples from 2015 to 2017, respectively, and was 14% for routinely sampled chicken and 18% for chicken cecal samples (2017). Our data found DSC of 40.4% (21 of 52) of the *Salmonella* isolates. Again, our results are notably worse. In our data, resistance in *Salmonella* to azithromycin was 0%. NARMS also did not find resistance to azithromycin in any chicken *Salmonella* isolates. Our results differ from NARMS data collected during the same time frame, and in some cases differ greatly. The differences may be due to the types of samples tested and other methodological differences. 

In 2014, Consumer Reports published data on antibiotic resistance in retail chicken in the United States and also defined multidrug resistant isolates as ‘those that are resistant to three or more classes of drugs that they would normally be susceptible to’ [43]. Thirty-eight percent of *Salmonella* isolates were found to be MDR in 2014, and 13% of *Campylobacter* isolates were found to be MDR, compared to 48.08% and 0% in this study, respectively. 

The limited number of isolates available for testing (16 samples for *Campylobacter* and 52 samples for *Salmonella*) greatly reduced what statistical analysis could discover beyond the overall frequencies of resistance to each drug. For *Campylobacter* isolates, nothing of statistical significance was identified. For the *Salmonella* isolates, no significant comparisons of interest were identified. 

## 4. Conclusions

Our testing at retail outlets reveals the antibiotic resistant bacteria that consumers are directly exposed to during handling, preparation and consumption of chicken, which is the most popular meat in the United States. A total of forty-eight percent of *Salmonella* isolates were resistant to three or more classes of antibiotics, suggesting that consumers may be exposed to multidrug resistant *Salmonella* through handling and consumption of raw or undercooked retail chicken breasts. Multidrug resistant *Salmonella* isolates included *Salmonella* Infantis as well as other serotypes, which have been implicated in various outbreaks. 

‘Farm-to-fork’ efforts, with distinction by poultry types and parts as well as strains, should continue to reduce *Salmonella* and *Campylobacter* contamination in chicken and control the development of antibiotic resistant strains. Consumers should continue to be encouraged to follow proper food safety practices when handling raw chicken, including proper storage, handling, and cooking temperature. At the regulatory level, the FDA could consider prohibiting antibiotic use in food animals except for therapeutic treatment. The USDA should consider classifying strains of *Salmonella* that are resistant to multiple antibiotics and known to have caused disease as “adulterants,” so that chickens tainted with those strains cannot be sold. 

## Figures and Tables

**Table 1:** *Salmonella* prevalence by regulatory category, 26 November 2017–24 November 2018 Data Set and 1 July 2018–29 June 2019 Data Set.

Regulatory *Salmonella* Category	*Salmonella* (Adjusted) Nov. 2017–2018 Data Set *		*Salmonella* (Adjusted) July 2018–June 2019 Data Set *	
3	A	19.70%	A	20.80%
2	B	2.50%	AB	8.40%
1	B	2.40%	B	1.90%

* Groups with different letters are statistically significantly different.

**Table 2:** Susceptibility patterns of *Salmonella* isolates to antibiotics used to treat infections in humans and resistance by number of classes.

Antibiotic/Number of Resistant Classes	Absolute Frequency of Resistant Isolates (*n* = 52)	Relative Frequency of Resistant Isolates (%)
Ciprofloxacin	0	NA *
Trimethoprim-sulfamethoxazole	9	17.31
Ceftriaxone	16	30.76
Azithromycin	0	NA *
Ampicillin	17	32.69
Resistant to 0 Antibiotics	12	23.07
Resistant to 1 class	5	9.62
Resistant to 2 classes	10	19.23
Resistant to 3 or more classes	25	48.08

* NA = Not Applicable.

**Table 3:** Susceptibility patterns of *Campylobacter* isolates to antibiotics used to treat *Campylobacter* infections in humans and resistance by number of classes.

Antibiotic/Number of Resistant Classes	Absolute Frequency of Resistant Isolates (*n* = 16)	Relative Frequency of Resistant Isolates (%)
Azithromycin	0	NA *
Ciprofloxacin	5	31.25
Resistant to 0 Antibiotics	6	37.5
Resistant to 1 class	8	50
Resistant to 2 classes	2	12.5
Resistant to 3 or more classes	0	NA*

* NA = Not Applicable.

## Data Availability

The data presented in this study are available on request from the corresponding author.

## Supplementary Materials

The following supporting information can be downloaded at: https://www.mdpi.com/article/10.3390/life13030642/s1, Table S1: Antibiotics Used for Susceptibility Testing of *Salmonella* Isolates. Table S2: Antibiotics Used for Susceptibility Testing of *Campylobacter* isolates. Table S3: *Salmonella* Isolates Resistant to more than one Recommended Antibiotic for Treatment of *Salmonella* Infection. Table S4: Antibiotic Susceptibility Testing Results for *Salmonella* and *Campylobacter* Isolates
